# Gun-Shot Wound of the Liver—Recovery

**Published:** 1859-06

**Authors:** E. M. Pendleton

**Affiliations:** Sparta, Georgia


### ARTICLE II.

*Gun-Shot Wound of the Liver—Recovery.* By E. M. PENDLETON, M. D., of Sparta, Georgia.

A negro boy named Henry, about twelve years of age, the property of William Barnsdale, was shot early in the month of December last, about two miles from this place. I was summoned to see him, in great haste, and arrived in about one hour after the occurrence. He was lying in much agony, and apparent apprehension of the result, from the anxiety depicted on his countenance. We ascertained that the ball entered in front, passing through the coat and shirt, about three inches below the right nipple, and on a parallel line with the epigastrium. It was discharged from a pistol, about three feet distance from his person, entered the intercostal space between the long and short ribs, inclining downwards and inwards. We proceeded to probe for the ball, (having learned none of the particulars,) when the patient informed us that it had passed through his body. On examination, we found this statement true. The ball had passed out just under the last short rib, and so near the spine as almost to graze it.

We proceeded to draw our prognosis of the case as best we might, which, from any point of view, was very unfavorable. Had the ball touched the lungs? No, for there was no cough, no disturbance of the respiratory functions, nor any other symptom indicative of pulmonary abrasion.

Was the diaphragm wounded? We thought not, because this must certainly have produced dyspnea, or at least, very laborious breathing. Nor could it have infringed upon the kidneys seriously, as it passed out a very little above that organ, although the night subsequent to the accident, the patient passed urine of a thick, muddy color, supposed to be tinged with blood by the nurse. The liver was the only organ that could have been wounded, as it lay direct in the range of the shot, and it must have passed directly through the upper part of that viscus.

As it has been asserted *ex cathedra,* that a man could not recover from a gun-shot wound, passing directly through the liver, thereby calling in question our diagnosis, we will examine a little more minutely into the reasons for this opinion, which, to our mind, amounts to a perfect demonstration, which the wiseacres and Sir Oracles in medicine may take for what they are worth. Let the medical reader, who knows at least, the great outlines of anatomy, place his finger three inches below his right nipple, a little outward, of a direct line, and then another of the other hand, just below the last rib, and very near the spinal column on the right side, and ask himself through what organ of the body would a ball have to pass from one of these points to the other. The unanimous voice would be the liver. Sir Oracle himself, gives it up, but objects, that, as the patient recovered, the ball must have struck a rib somewhat glancing, and run round it, coming out at the point designated. This is very cute certainly—calculated to mislead an uneducated mind, who knows just enough of the laws of motion, to see how such a thing might have happened. Unfortunately, however, for this sophism, the ribs do not run diagonally downwards, as would a line from one of these points to another, but laterally around the body; and if the word of the surgeon is not sufficient evidence to establish the fact, that the ball never touched a rib, any ignoramus would know that it was impossible for it to have run round one, though it had struck it, and come out three inches below the same rib on the opposite side.

In addition to this, the functions of the liver itself, were considerably disturbed. There was a paucity of bile in the excretions. The urine was of a whitish sedimentary character, notwithstanding the febrile excitement that supervened for several days. The pulse itself rose to 120 on the 3d day, and gradually subsided, under the antiphlogistic treatment instituted, as in the phlegmasia generally. Could a mere flesh wound of the supercostal integuments have excited and maintained such a pulse during a whole week? Certainly not. Nor could such a torpor of the liver supervene by which but little, if any, healthy bile passed either the bowels or the kidneys for several days. A slight acceleration of the pulse, with pain in the region of the liver, continued for several weeks, which finally passed off leaving the patient in his accustomed health.

We do not present this case as any thing remarkable in the annals of surgery, since more vital organs than the liver have been wounded without producing death—as the stomach, the lungs, the brain, and even the heart, but we felt it due to ourselves to vindicate our diagnosis of the case, which had been called into question, as well as to offer another somewhat remarkable instance of recovery from the passage of a leaden ball, almost centrally through the body.

